# Radioligand Therapy in Meningiomas: Today’s Evidence, Tomorrow’s Possibilities

**DOI:** 10.3390/cancers18020297

**Published:** 2026-01-18

**Authors:** Gabor Sipka, Kristof Apro, Istvan Farkas, Annamaria Bakos, Agnes Dobi, Katalin Hideghety, Laszlo Pavics, Sandor Dosa, Bence Radics, Marton Balazsfi, Pal Barzo, Melinda Szolikova, Zsuzsanna Besenyi

**Affiliations:** 1 Department of Nuclear Medicine and Theranostics, University of Szeged, 6720 Szeged, Hungary; sipka.gabor@med.u-szeged.hu (G.S.); bakos.annamaria@med.u-szeged.hu (A.B.);; 2 Department of Oncotherapy, University of Szeged, 6720 Szeged, Hungary; 3 Department of Pathology, University of Szeged, 6720 Szeged, Hungary; 4 Department of Neurosurgery, University of Szeged, 6720 Szeged, Hungary; 5 Mediso Ltd., 1037 Budapest, Hungary

**Keywords:** meningioma, Lu-DOTATATE, PRRT, theranostic

## Abstract

Meningiomas are the most common primary intracranial tumors, showing highly heterogeneous behavior and clinical outcomes. While the majority are benign, about one in five meningiomas are classified as higher grade (WHO Grade II–III), characterized by a more aggressive, treatment-resistant pathology. Although surgical resection remains the first-line therapy, peptide receptor radionuclide therapy is emerging as a novel and promising option for advanced, multifocal, or recurrent disease. The theranostic paradigm allows simultaneous detection and treatment of somatostatin receptor-expressing lesions using a single radiopharmaceutical. In this review, we explore the evolving role of PRRT in the management of meningiomas. We provide an integrated overview of preclinical findings—including radiosensitization mechanisms—and summarize the rapidly expanding clinical literature, which in recent years has grown both in patient numbers and in methodological sophistication. Particular emphasis is placed on advances in dosimetry, quantitative imaging, and radiomics, which are beginning to refine patient selection and improve response prediction. Together, current evidence highlights the therapeutic potential of radionuclide therapy in aggressive or refractory meningiomas and underscores the need for further prospective trials to define its optimal clinical application.

## Simple Summary

Meningioma is a tumor that arises from the membranes covering the brain. These lesions typically grow slowly, are often discovered incidentally, and may not cause any symptoms. When treatment is needed, surgery or radiotherapy are the most common options. However, in some cases, meningiomas can appear as multiple lesions, invade nearby bone, or develop into particularly aggressive and treatment-resistant forms that recur despite prior interventions. These more severe tumors are frequently associated with substantial clinical symptoms, such as epileptic seizures, and present major therapeutic challenges. In response to these difficulties, a rapidly evolving therapeutic approach has gained attention—radionuclide therapy, which delivers radioactive isotopes directly to tumor cells by targeting somatostatin receptors. Recent advances in molecular imaging, including PET- and SPECT-based receptor mapping, radiomics, and dose-based treatment planning, have further strengthened the biological rationale for this therapy. In this review, we summarize the current state of research on peptide receptor radionuclide therapy (PRRT) for meningioma.

## 1. Introduction

Meningioma is the most common primary intracranial tumor, with an incidence of approximately 8.8 per 100,000 individuals [1]. These tumors arise from the meningeal membranes covering the brain and spinal cord and typically exhibit slow, non-infiltrative growth. A substantial proportion of meningiomas are detected incidentally during neuroimaging performed for unrelated reasons, while others present with location-dependent clinical symptoms [2]. Common manifestations include headaches related to increased intracranial pressure, focal neurological deficits, cranial nerve dysfunction, and epileptic seizures resulting from cortical irritation or mass effect.

According to the World Health Organization (WHO) classification of tumors of the central nervous system, approximately 80% of meningiomas are classified as WHO grade I and are generally considered benign. The remaining cases are categorized as WHO grade II (atypical) or grade III (anaplastic), which are associated with increased mitotic activity, brain invasion, aggressive biological behavior, and markedly higher recurrence rates [3]. Typical biological behavior, recurrence risk, and somatostatin receptor expression patterns across WHO grades are summarized in Table 1. Despite advances in surgical techniques and radiotherapy, higher-grade meningiomas remain prone to recurrence even after apparently complete resection, underscoring the biological complexity of these tumors.

Beyond histopathological grading, meningiomas display pronounced inter-patient and intra-tumoral heterogeneity at the genetic, epigenetic, and cellular levels. Recurrent molecular alterations—including NF2 loss, TRAF7, KLF4, AKT1, SMO mutations, and dysregulation of the PI3K/AKT/mTOR pathway—define biologically distinct subgroups with variable growth patterns, recurrence risk, and therapeutic vulnerabilities [5]. Recent advances in molecular profiling and single-cell analyses have further highlighted the complexity of the meningioma tumor microenvironment, revealing dynamic interactions between neoplastic cells, immune infiltrates, and vascular components that may influence radiosensitivity and treatment resistance [5].

This molecular heterogeneity has important therapeutic implications. Multiple signaling pathways and oncogenic drivers have been proposed as potential treatment targets in meningioma, including angiogenic signaling via VEGF, immune checkpoint pathways, Hedgehog signaling, and PI3K/AKT/mTOR activation. An overview of the most relevant molecular targets and their corresponding therapeutic strategies is provided in Table 2. However, clinical translation of these targets has so far resulted in limited and inconsistent efficacy, and no systemic therapy has emerged as a broadly effective standard treatment for recurrent or refractory disease [6,7]. In contrast to many pathway-specific alterations that characterize only molecular subsets of meningioma, somatostatin receptor (SSTR) expression represents a unifying biological feature observed across the majority of tumors, largely independent of WHO grade or driver mutation status.

High-level expression of somatostatin receptors—particularly somatostatin receptor subtype 2 (SSTR2)—has been consistently demonstrated in meningiomas and constitutes one of their most distinctive molecular characteristics [8]. Importantly, SSTR expression is often preserved in recurrent or treatment-refractory disease, making it an attractive target even in advanced clinical settings. This biological feature provides the foundation for both receptor-based molecular imaging and targeted radionuclide therapy, forming the basis of the theranostic concept in meningioma management.

Peptide receptor radionuclide therapy (PRRT) exploits this molecular vulnerability by coupling somatostatin analogues to therapeutic radionuclides, enabling selective delivery of ionizing radiation to SSTR-expressing tumor cells. Unlike conventional external-beam radiotherapy, PRRT delivers continuous low-dose-rate irradiation at the cellular level, a property that may be particularly relevant for slowly proliferating but radioresistant tumor populations. The success of PRRT in neuroendocrine tumors has provided a strong biological and clinical rationale for its investigation in meningioma, particularly in patients with recurrent, inoperable, or radiation-refractory disease.

While surgery and radiotherapy remain the established cornerstones of meningioma management, a subset of patients ultimately exhausts standard local treatment options. In this context, SSTR-targeted radionuclide therapy has emerged as a biologically rational investigational approach. Diagnostic imaging strategies and evidence-based standard-of-care treatments are discussed in dedicated sections of this review.

The aim of the present narrative review is to synthesize current evidence on PRRT in meningioma from a translational perspective. We focus on the biological rationale underlying SSTR targeting, summarize relevant preclinical findings, critically evaluate the heterogeneous clinical literature, and discuss emerging strategies—including dosimetry-guided treatment and combination approaches—that may shape the future integration of PRRT into personalized meningioma therapy.

## 2. Diagnostics

Magnetic resonance imaging (MRI) remains the gold standard for the diagnosis and follow-up of meningiomas; however, when MRI is unavailable, computed tomography (CT) may be considered as an alternative. Conventional radiological imaging is essential for accurately assessing tumor extent, identifying distant intracranial foci, and evaluating peritumoral edema, bone involvement, and adjacent neurovascular structures. Meningiomas typically appear isointense on T1-weighted images and hyperintense on T2-weighted and fluid-attenuated inversion recovery (FLAIR) sequences, with strong and usually homogeneous contrast enhancement. To ensure precise evaluation and comparability across clinical trials, the use of standardized response assessment criteria—such as those defined by the Response Assessment in Neuro-Oncology (RANO) working group—is recommended [9].

As previously mentioned, meningiomas frequently express somatostatin receptors (SSTRs) on their cell surface, predominantly somatostatin receptor subtype 2 (SSTR2), which can be visualized using SSTR-targeted radiopharmaceuticals. For positron emission tomography (PET), commonly used tracers include [^68^Ga]Ga-DOTATATE, [^68^Ga]Ga-DOTATOC, [^68^Ga]Ga-DOTANOC, and, most recently, [^18^F]F-SiTATE—a SiFAlin tagged [Tyr3]-octreotate—as well as [^64^Cu]Cu-DOTATATE [10]. Single photon emission computed tomography (SPECT) imaging can be performed using [^99m^Tc]Tc-EDDA-Hynic-TOC or [^111^In]In-Pentetreotide [11]. These techniques provide valuable functional information regarding receptor density, tumor extent, and spatial relationship to surrounding healthy tissue, and may be particularly useful for confirming recurrence in postoperative settings where conventional MRI can be challenging.

**Figure 1:**
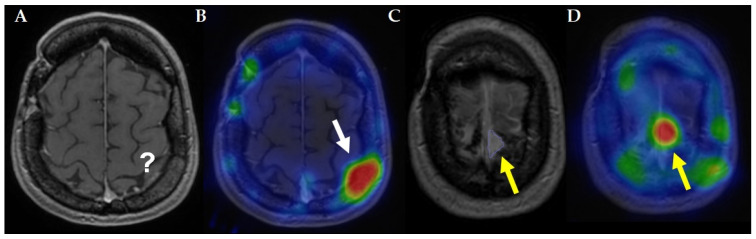
Representative comparison of contrast-enhanced MRI and somatostatin receptor-targeted SPECT in a patient with meningioma. (**A**) Contrast-enhanced MRI demonstrates no clearly detectable meningioma involvement of the cranial bone. (**B**) Corresponding SSTR SPECT image shows intense tracer uptake, indicating a somatostatin receptor-positive meningioma lesion (white arrow). (**C**) Contrast-enhanced MRI reveals an additional parafalcin foci in the precentral region, indicated by the yellow arrow. (**D**) SSTR SPECT confirms somatostatin receptor expression in the parafalcin lesion, demonstrating high tracer uptake at the corresponding site (yellow arrow). Images were acquired as part of routine clinical care and anonymized in accordance with institutional guidelines.

Due to minimal uptake in normal brain parenchyma, SSTR-targeted imaging offers superior lesion-to-background contrast, and high tracer accumulation in meningiomas has been shown to improve detection sensitivity compared with contrast-enhanced MRI alone [12]. The added value of SSTR-targeted imaging over conventional MRI is illustrated in Figure 1. In addition to its diagnostic value, SSTR imaging plays a pivotal role in identifying candidates for peptide receptor radionuclide therapy, as sufficient receptor expression is a prerequisite for treatment eligibility.

However, several important limitations of SSTR-based imaging must be considered. Meningiomas exhibit substantial intra-tumoral and inter-lesional heterogeneity in SSTR expression, which may result in heterogeneous tracer uptake within a single lesion or across multifocal disease. Moreover, SSTR expression may change over time, particularly following surgery or radiotherapy, highlighting the importance of contemporaneous molecular imaging when PRRT is being considered. False-positive uptake may also occur due to physiological SSTR expression in structures such as the pituitary gland, venous sinuses, or inflammatory tissue, particularly in anatomically complex skull base regions.

Importantly, tracer uptake intensity does not necessarily reflect intrinsic radiosensitivity, and high standardized uptake values (SUVs) alone do not guarantee therapeutic efficacy. These considerations underscore that SSTR imaging should be interpreted in conjunction with anatomical imaging, clinical presentation, and prior treatment history, rather than serving as a stand-alone decision-making tool.

Beyond lesion detection, molecular imaging also contributes to radiotherapy planning. Filippi et al. demonstrated that the integration of SSTR-targeted PET with MRI significantly improves lesion delineation, particularly for meningiomas located at the skull base or near the falx cerebri, thereby enabling more precise target volume definition and potentially reducing irradiation of adjacent critical structures [13].

Collectively, conventional MRI and SSTR-targeted molecular imaging provide complementary anatomical and biological information that is essential for accurate diagnosis, treatment planning, and patient selection. Ongoing developments in quantitative imaging, including volumetric assessment and advanced PET analysis, may further refine the diagnostic and predictive value of SSTR imaging in the future, particularly in the context of personalized radionuclide therapy.

## 3. Standard of Care

The management of meningiomas must be individualized, with the indication for treatment determined by tumor size, anatomical location, growth dynamics, and the presence or progression of neurological symptoms. While many incidentally detected or slowly growing meningiomas can be managed conservatively with active surveillance, intervention becomes necessary in the setting of documented tumor progression or the development of new or worsening neurological deficits, often related to critical tumor size or involvement of eloquent structures [4].

Surgical resection and radiotherapy remain the established, evidence-based cornerstones of meningioma treatment. Whenever feasible, maximal safe surgical resection is the preferred first-line approach, with long-term disease control strongly influenced by both the extent of resection and the underlying biological aggressiveness of the tumor. Despite advances in neurosurgical techniques and radiation delivery, meningiomas—particularly higher-grade lesions—remain prone to recurrence and progression, even after apparently optimal local treatment [14].

Radiotherapy plays a central role as an adjuvant or definitive treatment modality, especially in incompletely resected tumors, higher-grade meningiomas, or surgically inaccessible lesions. Modern radiotherapy techniques, including fractionated external-beam radiotherapy and stereotactic approaches, have improved local control while limiting toxicity to surrounding critical structures. Nevertheless, a clinically relevant subset of patients ultimately develops recurrent or progressive disease despite prior surgery and radiotherapy [4].

Importantly, even WHO grade I meningiomas can pose significant therapeutic challenges when located in anatomically complex regions such as the skull base or cavernous sinus, where surgical morbidity is high and complete resection is often not achievable. In patients who are no longer candidates for further surgical or radiotherapeutic intervention, treatment options become limited, and management decisions increasingly focus on disease stabilization and symptom control rather than curative intent [15].

In this setting, systemic therapies and other non-standard approaches have been explored; however, robust evidence supporting their efficacy remains lacking. To date, no systemic treatment has demonstrated consistent, practice-changing benefit in meningioma, and no randomized controlled trials have established an effective standard systemic therapy. Various targeted agents and multikinase inhibitors have been investigated, with reports of disease stabilization or occasional partial responses in small, non-randomized cohorts. In particular, inhibitors of vascular endothelial growth factor (VEGF) signaling have shown some potential to slow tumor progression in heavily pretreated patients, but responses are typically modest and transient. Overall, these approaches remain investigational and are generally considered palliative [16].

Taken together, current standard-of-care strategies for meningioma rely predominantly on local treatment modalities, while effective systemic options remain an unmet clinical need. This therapeutic gap has prompted increasing interest in biologically targeted approaches that exploit molecular features shared across meningioma subtypes. Among these, somatostatin receptor-directed strategies have gained particular attention and form the focus of the following section.

## 4. PRRT (Peptide Receptor Radionuclide Therapy)

Among the major innovations in nuclear medicine, theranostic concepts represent a paradigm shift by enabling individualized, target-specific therapeutic approaches that extend beyond conventional oncologic management. This strategy is based on the use of a diagnostic imaging tracer to visualize a tumor-specific molecular target in vivo, followed by administration of the same or an analogous molecule labeled with an α- or β-emitting radionuclide for therapeutic purposes. Somatostatin receptor-targeted theranostics have been successfully established in patients with neuroendocrine tumors and have subsequently been explored as a potential treatment option in meningioma [15,17].

At present, randomized clinical trials involving large patient cohorts are not available to evaluate the efficacy of PRRT in meningioma. Consequently, PRRT has not been approved by regulatory authorities such as the FDA or EMA for this indication and is currently considered an investigational treatment option. In the absence of meningioma-specific protocols, treatment regimens have largely been extrapolated from established neuroendocrine tumor protocols, a practice that introduces uncertainty regarding optimal dosing, cycle number, and patient selection [18].

Recommendations for the application of PRRT in meningioma are informed by joint guidance from the IAEA, EANM, RANO, and SNMMI, while patient selection criteria and radiopharmaceutical use are commonly based on the NANETS/SNMMI consensus statement [18,19]. It should be noted, however, that these recommendations are primarily derived from experience in neuroendocrine tumors and have not been prospectively validated in meningioma populations.

The most widely used radiopharmaceuticals for SSTR-directed radionuclide therapy are [^177^Lu]Lu-DOTATATE/DOTATOC and [^90^Y]Y-DOTATATE/DOTATOC. In patients with impaired renal function, [^177^Lu]Lu-DOTATATE is generally preferred due to its lower incidence of renal toxicity [20]. The commonly applied administered activities are 7.4 GBq for ^177^Lu-labeled compounds and 3.7 GBq for ^90^Y-labeled compounds, reflecting standard practice rather than meningioma-specific optimization.

The number of PRRT cycles typically ranges from two to five, with four cycles representing the most frequently applied regimen. Intervals between treatment cycles usually range from 6 to 12 weeks. If a cycle cannot be administered within the recommended timeframe, treatment may be postponed for up to 16 weeks without necessitating modification of the overall treatment plan. It should be emphasized that these dosing schemes and cycle numbers originate from neuroendocrine tumor practice and remain empirical in the context of meningioma.

In patients with relapsed or refractory meningiomas, PRRT should be considered only after confirmation of elevated somatostatin receptor expression by recent hybrid imaging (SPECT/CT or PET/CT), preferably within two months prior to treatment initiation, and the availability of a recent brain MRI, ideally obtained within two weeks. A Karnofsky performance status of at least 60% and an ECOG score not exceeding 2 are generally required. Baseline assessment of renal, hepatic, and bone marrow function is mandatory and should follow laboratory criteria outlined in the corresponding guidelines [18,21]. Discontinuation of long-acting somatostatin analogues and corticosteroids prior to PRRT is advised, as these agents may interfere with receptor targeting or downregulate SSTR expression [22].

Radiopharmaceuticals are typically administered via intravenous infusion. Two intravenous accesses are recommended—one for the radiopharmaceutical and one for the amino acid solution—although alternative approaches such as dual-chamber ports, alternating infusion through a single catheter, or intra-arterial administration may be considered in selected cases and are discussed elsewhere. Positively charged amino acids, such as L-lysine and/or L-arginine, are co-administered to mitigate renal toxicity by competitively inhibiting proximal tubular reabsorption of the radioligand [18,19]. Antiemetic prophylaxis, commonly with metoclopramide or ondansetron, is recommended prior to amino acid infusion and may be repeated if necessary. The radiopharmaceutical is typically administered with physiological saline over 10 to 30 min using gravity infusion, infusion pumps, or automated syringe systems.

All procedures must be conducted in compliance with local radiation safety regulations. Adequate hydration, reinforced personal hygiene measures, and continuous monitoring by qualified personnel are essential components of safe PRRT delivery.

Available evidence suggests that PRRT is generally well tolerated, with predominantly manageable and reversible adverse effects. While most toxicity data originate from large neuroendocrine tumor cohorts, smaller retrospective studies in meningioma patients report comparable safety profiles. According to the Common Terminology Criteria for Adverse Events (CTCAE), Grade 3 and 4 toxicities are mainly transient hematologic events, including neutropenia, thrombocytopenia, and lymphopenia, reported in approximately 1%, 2%, and 9% of patients, respectively, in large NET series [17]. The most frequently observed adverse events are Grade 1–2 nausea and vomiting, largely attributed to amino acid co-infusion, and typically resolve after completion of treatment.

In an ongoing prospective study evaluating [^177^Lu]Lu-DOTATATE in meningioma, adverse events were reported in approximately 10% of patients receiving four or more treatment cycles, with no Grade 4 toxicities confirmed by laboratory analysis [23].

Although encouraging safety profiles and signs of clinical activity have been reported in available PRRT studies involving refractory meningioma patients, comprehensive and methodologically homogeneous evidence remains limited [24]. This narrative review is therefore based on a structured literature search of PubMed, including preclinical studies, retrospective clinical series, prospective trials, and relevant consensus guidelines published up to 2025. Case reports and conference abstracts without full-text availability were excluded. Given the heterogeneity of the available data, the aim was not to provide an exhaustive systematic review, but rather a critical synthesis of biologically and clinically relevant evidence that prepares the ground for the subsequent preclinical and clinical sections.

## 5. Preclinical Studies

Preclinical investigations serve as the foundational basis for the development of medical therapies, and PRRT is no exception to this principle. In this section, the initial focus is placed on the available tumor models, which play a pivotal role in determining the translational relevance of radiobiological experiments. Following the description of the available tumor models, the subsequent sections will address various approaches to radiopharmaceutical modulation, including optimization strategies related to the choice of radionuclide (e.g., β- versus α-emitters), the targeting vector or pharmacophore, and improvements in the overall potency and stability of the compound. Preclinical studies have also explored the possibility of modifying the tumor microenvironment to enhance radionuclide uptake or therapeutic response. Finally, emerging combination strategies have been reported at the preclinical stage to improve PRRT efficacy, including the use of radiosensitizing agents, DNA repair inhibitors, immunotherapies, or integration with external beam radiation.

### 5.1. Meningioma Models

Meningiomas exhibit pronounced intra-tumoral and inter-patient heterogeneity at the cellular, transcriptional, and microenvironmental levels, posing a fundamental challenge for preclinical modeling. Recent human longitudinal single-cell and spatial transcriptomic analyses have demonstrated that individual meningiomas comprise multiple coexisting tumor cell states that can dynamically evolve over time, particularly during progression from primary to recurrent disease. These studies further reveal substantial variability in proliferative activity, metabolic programs, and DNA damage-response signatures both within and between tumors [25].

In parallel, the tumor microenvironment plays an active role in shaping tumor behavior, with recurrent meningiomas characterized by increased infiltration of immunosuppressive macrophage populations and complex tumor–stroma interactions. Together, these findings underscore that simplified in vitro or in vivo models only partially recapitulate the biological complexity of clinical meningiomas, an important consideration when interpreting preclinical PRRT data [25].

Within this broader preclinical framework, the availability and refinement of suitable meningioma models remain a decisive first step. Since PRRT currently plays only a limited role in meningioma therapy and is still under active investigation, preclinical models specifically tailored to PRRT research are particularly scarce. Nevertheless, a radiolabeled octreotide compound with high affinity for SSTR2 has long been available for rat studies, and mouse models of meningioma have also been developed [26,27]. Comparing the therapeutic effects of external beam radiation and radionuclide-based therapies remains a challenge in preclinical research. A recent study addressed this by employing in vitro, patient-derived meningioma spheroids to model PRRT efficacy. PRRT-induced DNA damage was detectable for a longer duration than with EBRT (External Beam Radiation Therapy), and the extent of DNA damage in the spheroids correlated with the level of SSTR2 expression in the original tumors. This model may serve as a valuable tool for future radiobiological studies assessing PRRT and EBRT responses [28]. It should be noted that, despite their utility, current meningioma models represent biologically reduced systems and may not fully capture the spatial heterogeneity, microenvironmental influences, and temporal evolution observed in human disease.

### 5.2. Radiopharmaceutical Modulation

Under the term radiopharmaceutical modulation, we refer to strategies aimed at optimizing the components and properties of the PRRT agent to improve targeting, efficacy, and safety. In the following sections, we will discuss these approaches in order: (1) choice of radionuclide (β- vs. α-emitters); (2) modifications of the targeting vector or pharmacophore; (3) improvements in stability, pharmacokinetics, and overall potency.

#### 5.2.1. Next-Generation Radionuclides for SSTR-Targeted Therapy

While current clinical research has largely focused on SSTR-targeted lutetium-based radiopharmaceuticals, emerging theranostic strategies are expanding the landscape of PRRT. One promising approach is the copper-based theranostic pair of ^64^Cu and ^67^Cu, which offers a compelling alternative to the conventional ^68^Ga/^177^Lu combination. The primary advantage of this approach lies in its potential for centralized production and large-scale distribution of ready-to-use radiopharmaceuticals. Preclinical studies have demonstrated the feasibility of [^67^Cu]-based PRRT in mouse models of pancreatic tumors, and early theranostic investigations have been initiated in other neuroendocrine tumors, with preliminary data on safety, biodistribution, and dosimetry also reported in cases of inoperable, multifocal meningiomas [29,30,31,32,33].

In parallel, ^161^Terbium has gained increasing attention as a potential therapeutic radionuclide. Its co-emission of numerous short-range electrons, including conversion and Auger electrons, delivers high linear energy transfer (LET), making it particularly effective against single cancer cells, especially when coupled with SSTR antagonists such as LM3, which bind to a larger number of receptor conformations on the cell surface and therefore enable higher tumor uptake and more homogeneous intratumoral dose delivery [34].

The α-emitting radionuclides offer further advantages over β-emitters due to their high LET and limited tissue penetration, enabling highly localized and potent tumor cell killing. This concept has already shown clinical promise in neuroendocrine tumors, as exemplified by the first-in-human [^212^Pb]Pb-DOTAMTATE trial [35]. These clinical findings are supported by preclinical studies with bismuth-213 and actinium-225 SSTR-targeted constructs [^213^Bi]Bi-DOTATATE, [^225^Ac]Ac-DOTA-LM3), which demonstrated robust anti-tumor efficacy and favorable safety profiles in neuroendocrine tumor models [36,37]. Collectively, these results highlight the potential of α-PRRT not only in neuroendocrine tumors but also in other SSTR-expressing tumors, such as meningiomas.

#### 5.2.2. Optimizing Targeted Ligands in Meningiomas

In this section, we focus on somatostatin receptors, their characteristics in meningiomas, and the molecular features that could serve as targets or catalysts for next-generation radionuclide therapies. The elevated expression of SSTRs in meningiomas is well documented [38]. While several receptor subtypes have been described, most data are derived from small case series; however, recent larger cohort studies provide more robust evidence [39,40]. Despite these advances, detailed information on the binding affinities of different SSTR subtypes and their potential contribution to radioresistance remains limited.

Overall, SSTR2 emerges as the predominantly expressed receptor in meningiomas, although SSTR1 and SSTR5 can also reach significant expression levels. Pharmacokinetic analyses using hybrid imaging further indicate that high radiopharmaceutical uptake correlates mainly with elevated SSTR2 expression and increased tumor vascularization, whereas SSTR3 and SSTR4 are typically low [41]. It is important to recognize that SSTR expression can change over time, and immunohistochemical findings from archived tumor samples may no longer reflect the receptor status in recurrent disease [40].

Over the past two decades, SSTR antagonists have been hypothesized to outperform agonists [42]. Accumulating evidence confirms that antagonists exhibit faster receptor binding, slower dissociation, and longer cellular retention, ultimately leading to higher tumor-absorbed radiation doses [43]. The SSTR antagonist [^177^Lu]Lu-DOTA-JR11 has already entered clinical trials with encouraging results, whereas [^177^Lu]Lu-DOTA-LM3 derivatives are currently in the preclinical phase [44,45].

Complementing these strategies, radiopharmaceutical modifications have been explored to improve stability, affinity, and tumor uptake. Notable examples include intraarterial [^177^Lu]Lu-HA-DOTATATE, which achieves nearly fourfold higher lesion uptake, and [^225^Ac]Ac-MACROPATATE, which demonstrates improved stability and slower degradation compared with conventional [^177^Lu]Lu-DOTATATE [46,47]. Similarly, the albumin-binding analog [^177^Lu]Lu-DOTA-EB-TATE shows prolonged systemic circulation and enhanced tumor retention, resulting in substantially higher absorbed doses than standard [^177^Lu]Lu-DOTATATE, further supporting the concept that pharmacokinetic engineering can enhance the efficacy of SSTR-directed therapies [48].

### 5.3. Microenvironment Modulation to Enhance PRRT

Epigenetic modulation of the tumor microenvironment can potentiate PRRT by increasing somatostatin receptor expression and enhancing radioligand binding. Histone deacetylase inhibitors (HDACis), for example, have been shown to upregulate SSTR2 transcription in neuroendocrine tumor models, leading to higher [^177^Lu]-DOTATATE uptake and improved therapeutic efficacy [49,50].

Beyond transcriptional effects, HDAC inhibitors such as valproic acid and vorinostat may also modify chromatin accessibility and microenvironmental features, a mechanism that remains biologically plausible but not yet experimentally confirmed in PRRT models.

### 5.4. Combination Therapies with PRRT

The integration of systemic therapies with peptide receptor radionuclide therapy represents a promising strategy for improving outcomes in aggressive or recurrent meningiomas, particularly in cases where PRRT monotherapy yields only modest responses [51]. Meningiomas display substantial biological heterogeneity, and several molecular and microenvironmental features contribute to their variable radiosensitivity. These include enhanced DNA-repair capacity, impaired apoptotic signaling, hypoxia, and activation of oncogenic survival pathways. Such mechanisms collectively limit the cytotoxic potential of radiation and provide a strong rationale for developing combination approaches that can amplify the biological effects of PRRT [5,51,52,53].

A major area of investigation focuses on targeting DNA damage-response pathways. PARP inhibitors impair single-strand break repair and can significantly increase the burden of unrepaired DNA lesions induced by β-particle irradiation [54,55]. Beyond PARP inhibition, ATM and ATR blockade may offer additional opportunities to augment DNA-damage accumulation [56]. Although their radiosensitizing effects have been convincingly demonstrated in external-beam radiotherapy models, evidence for their potentiation of β-particle radioligand therapy is still lacking. Preclinical evidence in meningioma models supports the susceptibility of these tumors to DNA-repair inhibition, and synergistic effects have been demonstrated in SSTR2-expressing neuroendocrine tumors treated with [^177^Lu]Lu-DOTATATE combined with talazoparib [57]. These findings suggest that mechanistically selected DNA-repair inhibitors could provide clinically meaningful radiosensitization in meningioma as well.

Modulation of the cell cycle represents another promising avenue. Preclinical studies show that gemcitabine increases radiation-induced DNA damage and growth suppression in malignant meningioma models [58], while similar radiosensitizing effects of agents such as temozolomide have been described in other CNS tumors [59]. Although these data derive from external-beam irradiation rather than PRRT, the radiobiological rationale and supportive preclinical findings justify further investigation.

Tumor hypoxia is a well-recognized contributor to radioresistance, and antiangiogenic treatments such as bevacizumab may help counteract this by temporarily improving tumor oxygenation [60,61]. Bevacizumab has also shown clinical benefit in high-grade meningioma, supporting its potential relevance alongside PRRT [62]. Additional radiosensitizing options described in the literature include chemotherapy agents that increase tumor vulnerability to radiation and strategies that modulate cell-death pathways activated by DNA damage, with recent findings in malignant meningioma models further highlighting the importance of p53-pathway-targeted approaches [63,64,65].

The molecular landscape of meningiomas provides further opportunities for rational combinations. NF2 loss frequently activates the PI3K/AKT/mTOR pathway, suggesting that mTOR inhibitors may augment PRRT-induced cytotoxicity by attenuating downstream survival signaling [66,67]. Similarly, BAP1-deficient tumors exhibit impaired chromatin remodeling and reduced DNA-repair capacity, potentially rendering them more susceptible to DNA-damaging approaches [68]. These molecular features support the development of precision-driven combination strategies tailored to tumor-specific vulnerabilities.

Finally, the intrinsic radiobiological properties of PRRT offer a unique therapeutic window for sustained radiosensitization. Because radiation-induced DNA damage occurs rapidly, whereas DNA-repair pathways remain active for 24–72 h, radiosensitizing agents are most effective when present before or during the early phase of ^177^Lu activity, allowing continuous low-dose-rate irradiation to suppress repair mechanisms [69,70,71]. A comparable rationale supports integrating external-beam radiotherapy (EBRT) as well: EBRT induces an acute surge of DNA damage and activates repair processes that may render tumor cells transiently more susceptible to the prolonged radiation delivered by PRRT [69,70,71]. The exceptionally high PFS-6 rates observed in the German prospective trials by Kreissl et al. and Hartrampf et al., although influenced by patient selection, are consistent with this concept and suggest that carefully timed EBRT→PRRT sequencing could amplify therapeutic efficacy [72,73]. Prospective studies are needed to determine how best to optimize timing, dosing, and safety for such combined approaches.

**Figure 2:**
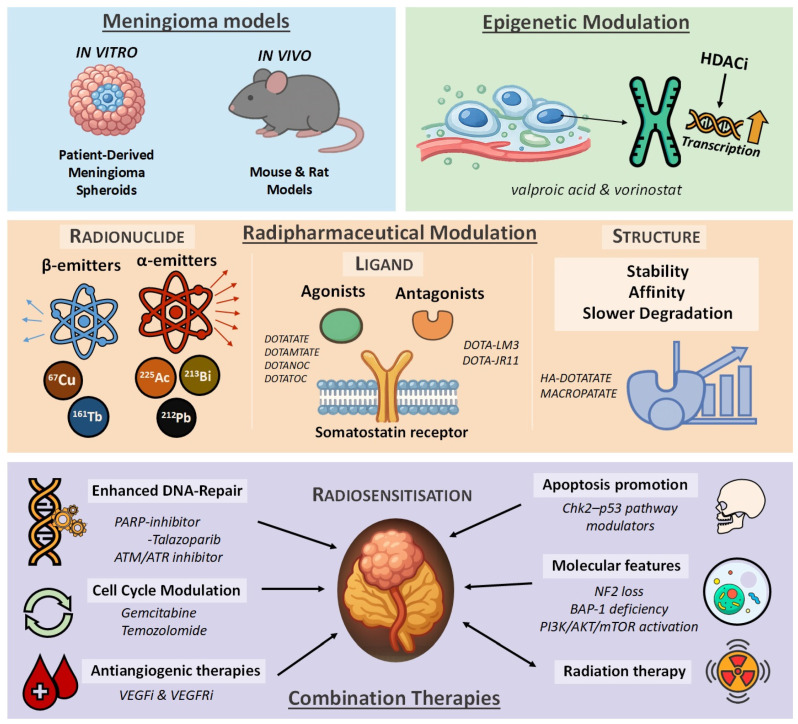
Summary diagram of preclinical studies in PRRT for meningioma. (HDACi: Histone deacetylase inhibitor; PARP: Poly(ADP-ribose) polymerase; ATM/ATR: Ataxia Telangiectasia Mutated/Ataxia Telangiectasia and Rad3-related inhibitors; VEGFi: Vascular Endothelial Growth Factor-inhibitor; VEGFRi: Vascular Endothelial Growth Factor Receptor-inhibitor; Chk2: Checkpoint kinase 2, NF2: Neurofibromatosis Type II.; BAP-1: BRCA1-associated protein 1; PI3K: Phosphatidylinositol 3-kinase; AKT: AKT Serine/Threonine Kinase 1; mTOR: Mammalian Target of Rapamycin).

Together, these combination strategies hold significant promise for overcoming intrinsic and acquired radioresistance in meningiomas (Figure 2). As clinical development progresses, biologically informed and molecularly stratified approaches will be essential for determining which tumors benefit from PRRT monotherapy and which require integrated systemic radiosensitization to achieve durable therapeutic responses.

Collectively, preclinical investigations of PRRT in meningioma provide a strong biological rationale for somatostatin receptor-targeted radionuclide therapy and identify multiple strategies to enhance its efficacy. At the same time, these studies are characterized by substantial heterogeneity in experimental models, endpoints, and translational relevance. Many findings are derived from simplified in vitro systems, animal models, or neuroendocrine tumor platforms that do not fully recapitulate the cellular complexity, microenvironmental interactions, and temporal evolution of human meningiomas. Consequently, the available preclinical evidence should be regarded as hypothesis-generating rather than directly practice-informing. Among the diverse approaches explored, radiosensitization strategies targeting DNA damage-response pathways and biologically informed combination concepts currently appear most amenable to clinical translation. These preclinical insights provide an essential framework for interpreting existing clinical data and for guiding the design of future translational and clinical studies, which are discussed in the following section.

## 6. Clinical Studies

### 6.1. Current Clinical Evidence and Study Landscape

Clinical experience with peptide receptor radionuclide therapy in meningiomas has expanded considerably over the past two decades, generating a growing body of evidence that supports its use in patients with recurrent or progressive disease refractory to surgery and external-beam radiotherapy. Although PRRT is not yet an established standard of care, its increasingly consistent performance across prospective and retrospective studies highlights its therapeutic potential in this challenging population. Importantly, the majority of available data are derived from small, non-randomized cohorts with heterogeneous patient populations, and should therefore be interpreted as hypothesis-generating rather than practice-changing.

Early investigations primarily used [^90^Y]Y-DOTATOC, particularly for large tumor volumes due to the higher-energy β-emission of yttrium, but after the success of the NETTER-1 trial in neuroendocrine tumors, [^177^Lu]Lu-DOTATATE became the dominant radiopharmaceutical owing to its favorable safety profile and more predictable dosimetry [17,74,75,76]. Consequently, most contemporary studies now focus on lutetium-based agents, improving comparability across cohorts.

A key challenge in synthesizing the PRRT literature is the heterogeneity of available studies, which vary in radiopharmaceutical choice, imaging methods, administered activity, response criteria, and patient selection. Clinical cohorts frequently include mixed WHO grades, variable prior surgical and radiotherapeutic histories, and differing definitions of progression, all of which introduce unavoidable sources of bias when interpreting outcomes.

### 6.2. Response Assessment and the Role of PFS-6

To address this variability, progression-free survival at six months (PFS-6) has become a central outcome measure. The RANO group recommends PFS-6 thresholds of >67% for WHO grade I and >49% for WHO grade II–III meningiomas as indicators of meaningful therapeutic activity [77]. These benchmarks provide a practical framework for interpreting treatment efficacy and allow PRRT outcomes to be contextualized against historical standards.

Across published clinical studies, complete or partial radiological responses remain relatively uncommon; however, disease stabilization is frequently observed, yielding high overall disease control rates (Table 3). Most cohorts report PFS-6 values between 60–70%, surpassing RANO efficacy thresholds even among heavily pretreated patients. While this consistency is noteworthy, disease stabilization and PFS-6 must be interpreted with caution in meningioma, a typically slow-growing tumor entity in which short-term progression-free endpoints may overestimate therapeutic benefit, particularly in non-randomized and uncontrolled settings.

No clear efficacy differences have been demonstrated between lutetium- and yttrium-based therapies, and most studies administered four treatment cycles with cumulative activities remaining below 30 GBq. These findings align with the currently preferred regimen of four cycles of 7.4 GBq [^177^Lu]Lu-DOTATATE, which appears to provide an optimal balance between antitumor activity and tolerability [18].

### 6.3. Key Study Results and Clinical Interpretation

Several studies stand out due to their methodological strengths or notable outcomes. Marincek et al. and Seystahl et al. contributed foundational evidence, demonstrating high rates of disease stabilization with both [^90^Y]Y- and [^177^Lu]Lu-labeled somatostatin analogues [21,78]. Minczeles et al. reported durable progression control across a mixed-grade cohort, while Severi et al. observed some of the most favorable PFS-6 outcomes in the literature, underscoring the importance of standardized response assessment and careful patient selection [79,80]. Mirian et al. provided the largest prospective dataset of [^177^Lu]Lu-DOTATATE monotherapy to date, identifying factors such as SSTR uptake intensity and prior radiotherapy as potential predictors of benefit [24]. More recently, aggregated analyses—including a 2025 meta-analysis by Zhang et al. and the 2025 pooled evaluation by Muoio et al.—have reinforced PRRT’s clinical utility, reporting disease control rates around 60–66% in real-world, heterogeneous populations [81,82]. However, the inherent variability of included studies and the predominance of retrospective data underscore the need for cautious interpretation of pooled efficacy estimates.

Only two prospective studies to date—those by Kreissl et al. and Hartrampf et al.—combined PRRT with external-beam radiotherapy [72,73]. These investigations demonstrated remarkably high PFS-6 values, although their interpretation requires caution because the included patients largely had WHO grade I and II tumors that were inoperable but not treatment-refractory. Nonetheless, the results highlight the biological plausibility and promising clinical potential of dual-radiation strategies, which are increasingly supported by preclinical radiosensitization data.

Taken together, the studies summarized in Table 3 reveal several consistent patterns despite substantial heterogeneity in study design and patient populations. Across cohorts, typical PFS-6 values range from approximately 60–70%, with higher rates generally observed in WHO grade I tumors and lower—but frequently still RANO-exceeding—rates in WHO grade II and III disease. Objective radiological responses (complete or partial responses) are rare, and clinical benefit is predominantly driven by disease stabilization rather than tumor regression. Notably, no clear or consistent differences in efficacy have emerged between yttrium- and lutetium-based radionuclides or across retrospective versus prospective single-arm studies, suggesting that observed treatment effects are relatively robust across different PRRT platforms and clinical settings.

### 6.4. Emerging Trends and Novel Approaches

Important methodological innovations have also emerged. Intra-arterial PRRT, investigated by Puranik et al. and Amerein et al., seeks to enhance radiopharmaceutical delivery by bypassing the blood–tumor barrier [46,84]. While early findings are encouraging, a clear superiority over conventional intravenous administration has yet to be demonstrated.

New radiopharmaceuticals further enrich the therapeutic landscape. [^177^Lu]Lu-HA-DOTATATE, with high affinity for SSTR2 and SSTR5, has transitioned from preclinical evaluation to clinical testing and may provide similar efficacy with improved tolerability [46]. Somatostatin receptor antagonists represent an especially promising frontier: phase 0 data for [^177^Lu]Lu-DOTA-JR11 indicate 2–5× higher tumor absorbed doses and a superior therapeutic index compared with traditional agonists, despite requiring substantially lower administered activities [44]. These features could prove particularly advantageous in radioresistant molecular subtypes.

Beyond clinical outcomes themselves, the integration of quantitative imaging and dosimetry has become increasingly essential for interpreting PRRT efficacy and for guiding individualized treatment strategies [88,89,90]. Increasing standardization in imaging techniques and response assessment has significantly improved study comparability. Earlier trials used heterogeneous MRI protocols, CT imaging, or non-uniform PET & SPECT tracers, and often lacked consistent RANO-based evaluation. More recent studies increasingly apply RANO meningioma criteria, enabling more reliable cross-trial synthesis and supporting reproducible clinical endpoints [88,91]. As quantitative SSTR PET or SPECT metrics and radiomic biomarkers continue to evolve, standardized imaging is expected to play a growing role in patient selection and response prediction [92,93,94].

Recent advances in imaging and dosimetry are beginning to transform how PRRT is evaluated and optimized in meningioma. Traditionally, response monitoring relied mainly on anatomical MRI and semiquantitative PET parameters such as SUVmax, which offer limited insight into intratumoral heterogeneity and radiation sensitivity. Modern quantitative imaging methods—including individualized post-therapy dosimetry and radiomics—now allow a more detailed assessment of radiopharmaceutical distribution and tumor biology, supporting more refined treatment stratification [88,89,95,96,97].

Personalized dosimetry is becoming a central component of modern PRRT, partly because radiopharmaceutical uptake in intracranial tumors, including meningiomas, is often heterogeneous. Experience from neuroendocrine tumor studies shows that post-therapy SPECT/CT can reveal marked variation in absorbed dose across tumor subregions, with some “cold areas” receiving subtherapeutic radiation despite adequate overall uptake [89,90,95]. Higher absorbed doses have consistently been associated with improved response and longer progression-free survival in NET-PRRT, supporting the rationale for shifting from fixed-activity regimens toward dose-guided, individualized planning [90,98,99]. Although dose–response relationships have not yet been formally established in meningioma, the shared SSTR-targeting mechanism and radiobiological principles provide a mechanistically sound basis for further investigation [98].

Radiomics is also emerging as an important tool for characterizing biological heterogeneity relevant to PRRT response. In meningioma, radiomic signatures derived from MRI and SSTR-PET have shown promise in grading, risk stratification, and identifying aggressive phenotypes. Studies in neuro-oncology and SSTR-expressing tumors indicate that texture-based radiomic features—such as entropy, uniformity, and grey-level heterogeneity—may outperform simple SUV-based metrics in capturing biologically relevant variation [100,101]. Radiomics applied to post-therapy SPECT/CT imaging is an emerging concept, but could theoretically provide even more predictive value by reflecting the true therapeutic distribution rather than receptor density alone [95,101].

Together, individualized dosimetry and radiomics represent key steps toward precision radionuclide therapy, enabling biologically informed treatment adaptation, improved patient selection, and potentially better identification of candidates for combination strategies. As imaging methodologies continue to standardize—driven by broader use of RANO meningioma response criteria—future clinical trials are expected to integrate these quantitative tools more systematically [88,91,92].

Finally, the field is now approaching a pivotal moment with the launch of LUMEN-1 (NCT06326190), the first randomized clinical trial evaluating [^177^Lu]Lu-DOTATATE in refractory meningioma. While LUMEN-1 represents a critical step toward higher-level evidence, its results should currently be regarded as hypothesis-defining rather than practice-changing, with the potential to clarify optimal timing, patient selection, and the future role of PRRT within multimodality meningioma treatment [102].

## 7. Conclusions

In summary, the growing body of clinical, translational, and preclinical evidence positions PRRT as a biologically rational and increasingly impactful therapeutic option for patients with recurrent or progressive meningioma. Across contemporary studies, PRRT demonstrates consistent disease control rates, favorable long-term tolerability, and mechanistic relevance rooted in SSTR-targeted delivery and sustained radiation. While current clinical evidence remains largely derived from heterogeneous and non-randomized cohorts, the reproducibility of observed disease stabilization across different study designs supports the continued investigation of PRRT in carefully selected patient populations. Together with emerging insights from dosimetry, quantitative imaging, and tumor biology, these findings suggest that PRRT represents a maturing investigational modality rather than a purely experimental salvage strategy. Continued clinical development will be essential to refine patient selection, optimize administered activity and treatment sequencing, and define the therapeutic settings in which PRRT can offer the greatest clinical benefit within multimodality meningioma management.

## 8. Future Directions

Building on the current evidence base, several avenues of investigation are now poised to reshape how PRRT is integrated into meningioma care. As molecular profiling becomes increasingly embedded in clinical workflows, meningiomas are likely to be stratified into biologically distinct subgroups defined by their inherent radiosensitivity and treatment susceptibility [16,103]. In this context, tumors with favorable radiobiological characteristics—such as limited DNA-repair capacity or preserved apoptotic signaling—may represent suitable candidates for PRRT monotherapy, particularly when guided by individualized dosimetry and quantitative SSTR imaging. Such an approach aligns with emerging trends in other SSTR-expressing tumors, where ongoing trials such as NETTER-3 (NCT06784752) are now investigating whether earlier-line implementation of PRRT can improve clinical outcomes—an approach that, if validated, may hold similar promise for molecularly selected meningioma populations [104].

Conversely, radioresistant meningiomas—driven by mechanisms such as DNA-repair upregulation, hypoxia, or activation of the PI3K/AKT/mTOR pathway—will likely require combination approaches to achieve meaningful disease control. Rationally selected radiosensitizers, including DNA-damage response inhibitors, cell-cycle modulators, and hypoxia-targeting agents, may amplify the cytotoxic effects of PRRT when administered within the 24–72 h window of maximal radionuclide-induced DNA injury. For tumors harboring specific pathway alterations, targeted agents such as mTOR or PI3K/AKT inhibitors may further enhance treatment efficacy by mitigating survival signaling and promoting apoptotic susceptibility.

Future clinical trials will need to integrate molecular, imaging-based, and dosimetric biomarkers to build predictive frameworks that distinguish patients who benefit most from PRRT monotherapy from those requiring combination therapy. Additionally, comparative evaluation of emerging radiopharmaceutical platforms—including α-emitters, copper-based theranostic pairs, and high-affinity SSTR antagonists—may expand therapeutic opportunities and improve tumor dose delivery in challenging disease subsets. Key ongoing and clinically investigated PRRT-based strategies in meningioma are summarized in Table 4, while detailed outcome data from completed studies are discussed in the Clinical Studies section.

Ultimately, progress in this field will depend on harmonized, prospective multicenter studies designed to evaluate optimized dosing strategies, treatment sequencing, combination regimens, and long-term clinical outcomes. As evidence continues to mature, PRRT has the potential to evolve from a later-line investigational option toward a biologically tailored component of personalized meningioma therapy, applied according to tumor-specific molecular vulnerabilities and radiobiological behavior. While these strategies remain investigational, their integration into prospective trial design will be critical to determine their true clinical value.

## Figures and Tables

**Table 1:** WHO grading, biological behavior, recurrence risk, and somatostatin receptor expression in meningioma.

WHO Grade	Biological Behavior	Approximate 5-Year Recurrence Rate *	Typical SSTR Expression
Grade I	Benign, slow-growing	7–23%	High, predominantly SSTR2
Grade II	Atypical, invasive	50–55%	High to moderate, mainly SSTR2
Grade III	Anaplastic, aggressive	72–78%	Variable but frequently preserved SSTR2

* Recurrence rates after gross total resection [4].

**Table 2:** Summary of the most important molecular targets of meningioma and the associated therapeutic options [4].

Molecular Targets	Common Grade	Drug Class
AKT1 mutation	Grade I	AKT inhibitor
SMO mutation	Grade I	Hedgehog inhibitor
NF2 loss	All Grades	FAK inhibitor
PD-L1, PD-L2, CTLA-4	Grade II and III	Immune checkpoint inhibitor
VEGF or VEGFR2	Grade III	VEGF or VEGFR inhibitor
PI3K	Grade II	PI3K inhibitor
mTOR	Grade II and III	mTOR inhibitor
Cytidine	Grade II and III	Gemcitabine
Somatostatin receptor	All Grades	Somatostatin analog or PRRT

**Table 3:** Summary table of the most important clinical publications on radionuclide therapy used in patients with meningioma. (PFS: Progression-Free Survival; SD: Stable Disease; CR: Complete Response; PR: Partial Response; PD: Progressive Disease; EBRT: External Beam Radiation Therapy; SWOG: Southwest Oncology Group; RECIST: Response Evaluation Criteria in Solid Tumors; RANO: Response Assessment in Neuro-Oncology, DCR: Disease Control Rate. N/A: not available).

Author	Year	Type of Study	Meningioma (Total Cohort)	Therapy	Cycles (Activity)	Response	All Grade PFS 6 Month %	Median PFS (Months)	Radiological Criteria for Progression
Marincek et al. [21]	2015	Prospective	*n* = 34	^90^Y-DOTATOC ^177^Lu-DOTATOC	1–4 (1.5–22.2 GBq)	SD = 23 (67.6%) PD = 11 (32.4%)	N/A	N/A	RECIST 1.1
Merrel et al. [23]	2024	Prospective	*n* = 20	^177^Lu-DOTATATE	4 (29.6 GBq)	N/A	77.8%	10.7	RANO
Mirian et al. [24]	2021	Meta-analysis	*n* = 111	^111^In-Pentetreotide ^90^Y-DOTATOC ^177^Lu-DOTATOC ^177^Lu-DOTATATE	1–4 (1.7–29.8 GBq)	PR = 2 (1.8%) SD = 64 (57.7%) PD = 45 (40.5%)	61.0%	Not reached	SWOG RECIST 1.1 Macdonald
Eigler et al. [44]	2024	Prospective	*n* = 6	^177^Lu-DOTATOC + ^177^Lu-DOTA-JR11	3–4 (14.1–18.9 GBq)	SD = 5 (83.3%) PD = 1 (16.7%)	N/A	N/A	Volume Imaging Criteria
Amerein et al. [46]	2024	Retrospective	*n* = 13	^177^Lu-HA-DOTATATE	1–4 (7.5–30.1 GBq)	CR = 1 (7.7%) PR = 1 (7.7%) SD = 8 (61.5%) PD = 3 (23.1%)	76.9%	18	RANO
Kreissl et al. [72]	2012	Prospective	*n* = 10	EBRT + ^177^Lu-DOTATATE ^177^Lu-DOTATOC	1 (7.4 ± 0.3 GBq)	CR = 1 (10%) PR = 1 (10%) SD = 8 (80%)	100.0%	N/A	N/A
Hartrampf et al. [73]	2020	Prospective	*n* = 10	EBRT + ^177^Lu-DOTATATE ^177^Lu-DOTATOC	1 (7.4 ± 0.3 GBq)	CR = 1 (10%) SD = 6 (60%) PD = 3 (30%)	100.0%	91.1	RANO
Bartolomei et al. [74]	2009	Prospective	*n* = 29	^90^Y-DOTATOC	2–6 (5–15 GBq)	SD = 19 (65.5%) PD = 10 (34.5%)	46.4%	61 (Grade I) 13 (Grade II–III)	SWOG
Minutoli et al. [75]	2014	Retrospective	*n* = 8	^111^In-Pentetreotide ^90^Y-DOTATOC ^177^Lu-DOTATATE	2–4 (4.8–29 GBq)	PR = 2 (2%) SD = 5 (62.5%) PD = 1 (12.5%)	N/A	N/A	SWOG
Gerster-Gillieron et al. [76]	2015	Prospective	*n* = 15	^90^Y-DOTATOC	2–4 (1.35–14.8 GBq)	SD = 13 (86.7%) PD = 2 (13.3%)	86.7%	24	RECIST 1.1
Seystahl et al. [78]	2016	Retrospective	*n* = 20	^90^Y-DOTATOC ^177^Lu-DOTATATE	1–4 (3.4–27.6 GBq)	SD = 10 (50%) PD = 10 (50%)	42.0%	5.4	Macdonald
Minczeles et al. [79]	2023	Retrospective	*n* = 15	^177^Lu-DOTATATE	1–4 (7.5–30.5 GBq)	SD = 6 (40%) PD = 9 (60%)	60.0%	7.8	RANO
Severi et al. [80]	2024	Prospective	*n* = 42	^90^Y-DOTATOC ^177^Lu-DOTATATE	2–6 (6.6–33 GBq)	PR = 1 (2.4%) SD = 23 (54.8%) PD = 18 (42.8%)	75.0%	16	RANO
Zhang et al. [81]	2025	Retrospective	*n* = 18	^177^Lu-DOTATATE ^90^Y-DOTATOC ^177^Lu-HA-DOTATATE	2–4 (8.5–28.5 GBq)	SD = 12 (85.7%) PD = 2 (14.3%)	N/A	32.3	RANO
Muoio et al. [82]	2025	Meta-analysis	*n* = 269	^111^In-Pentetreotide ^90^Y-DOTATOC ^177^Lu-DOTATOC ^177^Lu-DOTATATE	1–4 (7.1–29.6 GBq)	PR ≈ 2% SD ≈ 65% PD ≈ 33%	N/A (DCR = 67.7%)	10–18%	SWOG RECIST 1.1 Macdonald RANO Volume Imaging Criteria
Van Essen et al. [83]	2006	Retrospective	*n* = 5	^177^Lu-DOTATATE	2–4 (14.8–29.6 GBq)	SD = 2 (40%) PD = 3 (60%)	40.0%	N/A	SWOG
Puranik et al. [84]	2024	Retrospective	*n* = 8	^177^Lu-DOTATATE	2–4 (14.8–29.6 GBq)	SD = 2 (25%) PD = 8 (75%)	N/A	8.9	RANO
Parghane et al. [85]	2019	Retrospective	*n* = 5	^177^Lu-DOTATATE	2–6 (13.28–29.97 GBq)	SD = 5 (100%)	100.0%	26.3	RECIST 1.1
Müther et al. [86]	2020	Retrospective	*n* = 7	^177^Lu-DOTATATE	2–4 (14.8–29.6 GBq)	SD = 1 (14.3%) PD = 6 (85.7%)	42.9%	N/A	RANO
Kertels et al. [87]	2021	Retrospective	*n* = 11	^177^Lu-DOTATATE ^177^Lu-DOTATOC	2–6 (6.9–29.6 GBq)	SD = 6 (54.5%) PD = 5 (45.5%)	N/A	12	RANO
Salgues et al. [88]	2022	Retrospective	*n* = 8	^177^Lu-DOTATATE	2–4 (10.6–29.6 GBq)	SD = 7 (87.5%) PD = 1 (12.5%)	85.7%	N/A	RANO

**Table 4:** Key ongoing and clinically investigated PRRT-based strategies in meningioma [44,46,72,73,102].

Study/Strategy	Population	PRRT Strategy	Clinical Status/Focus
**LUMEN-1** (NCT06326190)	Refractory WHO I–III meningioma	[^177^Lu]Lu-DOTATATE vs. standard care	Ongoing randomized phase II efficacy trial
**EBRT + PRRT sequencing** (no NCT; Kreissl et al., Hartrampf et al.)	Inoperable WHO I and II meningioma	External-beam radiotherapy followed by PRRT	Completed prospective clinical studies evaluating dual-radiation strategies
**Everolimus + PRRT** (NCT06126588)	Refractory WHO II and III meningioma	[^177^Lu]Lu-DOTATATE + everolimus	Ongoing phase IIb combination trial
**Intra-arterial PRRT** (exploratory clinical studies)	Recurrent/refractory meningioma	IA [^177^Lu]Lu-DOTATATE/[^177^Lu]Lu-HA-DOTATATE	Early clinical evaluation of enhanced tumor dose delivery
**SSTR antagonist PRRT (e.g., JR11)** (NCT04997317)	SSTR-positive meningioma	[^177^Lu]Lu-DOTA-JR11	Phase 0/I evaluation of therapeutic index
**Dosimetry-guided PRRT** (integrated in prospective protocols)	Recurrent meningioma	Personalized [^177^Lu]Lu-DOTA-TATE	Individualized activity planning and response optimization

## Data Availability

Data is contained within the article.

## Abbreviations

The following abbreviations are used in this manuscript:

WHO	World Health Organization
PRRT	Peptide Receptor Radionuclide Therapy
HGM	High Grade Meningioma
MRI	Magnetic Resonance Imaging
FLAIR	Fluid-Attenuated Inversion Recovery
CT	Computer Tomography
RANO	Response Assessment in Neuro-Oncology
SSTR	Somatostatin Receptor
PET	Positron Emission Tomography
SPECT	Single Photon Emission Computer Tomography
FDA	Food and Drug Administration
EMA	European Medicines Agency
IAEA	International Atomic Energy Agency
EANM	European Association of Nuclear Medicine
SNMMI	Society of Nuclear Medicine and Molecular Imaging
NANETS	North American Neuroendocrine Tumor Society
CTCAE	Common Terminology Criteria for Adverse Events
ECOG	Eastern Cooperative Oncology Group
DNA	Deoxyribonucleic Acid
EBRT	External Beam Radiation Therapy
LET	Linear Energy Transfer
PARP-1	Poly(ADP-ribose) polymerase 1
AKT-1	AKT Serine/Threonine Kinase 1
SMO	Smoothened receptor
NF2	Neurofibromatosis type 2
FAK	Focal Adhesion Kinase
PD-L1	Programmed death-ligand 1
PD-L2	Programmed death-ligand 2
CTLA-4	Cytotoxic T-lymphocyte-associated protein 4
VEGF	Vascular Endothelial Growth Factor
VEGFR	Vascular Endothelial Growth Factor Receptor
PI3K	Phosphatidylinositol 3-kinase
PI3KCA	Phosphatidylinositol-4,5-bisphosphate 3-kinase catalytic subunit alpha
mTOR	Mammalian Target of Rapamycin
Chk-2	Checkpoint kinase 2
p53	p53 tumor suppressor protein
BAP1	BRCA1-associated protein 1
NETTER-1	Neuroendocrine Tumors Therapy-1 Trial
PFS	Progression-Free Survival
CR	Complete Response
PR	Partial Response
SD	Stable Disease
PD	Progressive Disease
SWOG	Southwest Oncology Group
RECIST	Response Evaluation Criteria in Solid Tumours
DCR	Disease Control Rate
CNS	Central Nervous System
